# Perceived Life Expectancy Is Associated with Colorectal Cancer Screening in England

**DOI:** 10.1007/s12160-016-9855-z

**Published:** 2016-11-07

**Authors:** Lindsay C. Kobayashi, Christian von Wagner, Jane Wardle

**Affiliations:** 1 Center for Population and Development Studies, Harvard T.H. Chan School of Public Health, Harvard University, 9 Bow St, Cambridge, MA 02138 USA; 2 Health Behaviour Research Centre, Department of Epidemiology and Public Health, University College London, London, UK

**Keywords:** Colorectal cancer, Cancer screening, Fecal occult blood test, Perceived life expectancy, Behavioral economics

## Abstract

**Background:**

Cancer screening is a behavior that represents investment in future health. Such investment may depend on how much ‘future’ a person expects.

**Purpose:**

The purpose of this study was to investigate the prospective association between perceived personal life expectancy and participation in fecal occult blood test screening for colorectal cancer (CRC) in a national program.

**Methods:**

Data were from interviews with 3975 men and women in the English Longitudinal Study of Ageing (ELSA) within the eligible age range for the national screening program (60 to 74 years). Perceived life expectancy was indexed as the individual’s estimate of their chance of living another 10–15 years (exact time varied by age), assessed in 2008/2009. Participation in CRC screening from 2010 to 2012/2013 was assessed in 2012/2013. Logistic regression was used to estimate the association between perceived life expectancy and screening participation, adjusted for numeracy and known mortality risk factors.

**Results:**

Overall, 71% of respondents (2817/3975) reported completing at least one fecal occult blood test (FOBt) during the follow-up. Screening uptake was 76% (1272/1683) among those who estimated their 10–15-year life expectancy as 75–100%, compared with 52% (126/243) among those who estimated theirs as 0–25% (adjusted OR 1.74, 95% CI 1.29–2.34).

**Conclusions:**

A longer perceived life expectancy is associated with greater likelihood of participating in CRC screening in England. However, half of people with a low perceived life expectancy still participated in screening. Given that CRC screening is recommended for adults with a remaining life expectancy of ≥10 years, future research should investigate how to communicate the aims of screening more effectively.

## Introduction

Colorectal cancer (CRC) is the third most common cancer in men and the second most common cancer in women worldwide, with an estimated 1.4 million cases and 693,300 deaths in 2012 [1]. The highest incidence rates are in developed countries, many of which have screening tests available through opportunistic or organized programs [2]. In the UK, the National Health Service (NHS) provides CRC screening without charge using the fecal occult blood test (FOBt), which is mailed biennially to the home addresses of all men and women aged 60–74 years. Although the NHS Bowel Cancer Screening Programme was one of the first organized CRC screening programs worldwide, the UK lags behind other countries internationally in CRC outcomes, making effective screening an important public health priority [3]. However, screening uptake is low, with just over 50% of eligible adults participating in any biennial screening round. In other countries where FOBt screening is offered, uptake also tends to be low [2]. Because routine screening improves CRC outcomes and reduces mortality in the population [4, 5], a better understanding of the causes of low uptake is needed to inform system-level interventions to increase participation.

A potentially salient factor in explaining cancer screening uptake that has been previously overlooked is perceived life expectancy (PLE). Participation in cancer screening represents an investment in future health. Screening participants undergo the immediate burden of completing a screening test in the hope of protecting their future health and survival through a negative result or an early diagnosis of cancer. This perspective on health, as a form of human capital that depreciates with time and can be invested in through behaviors such as cancer screening, forms the basis of the theory of health as human capital [6–8]. PLE estimates are informed by knowledge that people have readily at hand that may not always be queried about in study questionnaires, such as a more complete range of personal health and risk behaviors. PLE is predictive of actual mortality in American adults aged 50 and over in the US Health and Retirement Study across 2- and 8-year follow-up periods [9, 10]. The utility of PLE for predicting future behavior and actual mortality risk has been advocated for in the economic literature [11].

It is important to understand the nature of any potential association between PLE and CRC screening uptake, as the American College of Physicians does not recommend cancer screening for adults who have a remaining life expectancy of less than 10 years [12]. PLE could therefore be a useful factor that people may take into account when making informed decisions about cancer screening, but little is known about the degree to which it influences these decisions. Previously, PLE has been positively associated with uptake of mammography screening for breast cancer in the European Survey of Health, Ageing, and Retirement (SHARE), where women who expected to live longer were more likely to take up screening than those with low expectations of their future longevity [13]. The prospective association between PLE and participation in CRC screening through a nationally organized screening program is unknown.

We aimed to investigate the prospective association between perceived life expectancy, indexed as an individual’s own estimate of their chance of living another 10–15 years in 2008/2009, and participation in CRC screening through England’s NHS Bowel Cancer Screening Programme from 2010 to 2012/2013. We hypothesized that participation in CRC screening, a form of future health investment, would be higher among those with higher self-perceived remaining life expectancy.

## Materials and Methods

### Sample

The English Longitudinal Study of Ageing (ELSA) is a population-based cohort study of English men and women aged ≥50 years [14]. The ELSA cohort was established in 2002 and is based on a random stratified sample of English households [14]. The “core” ELSA sample consists of respondents recruited in the original 2002 sample (*n* = 12,100; response rate = 66%) and newer respondents recruited at subsequent waves of data collection to account for the aging structure of the original sample. ELSA data are collected in biennial “waves” through computer-assisted in-person interviews in the study respondents’ homes.

**Fig. 1:**
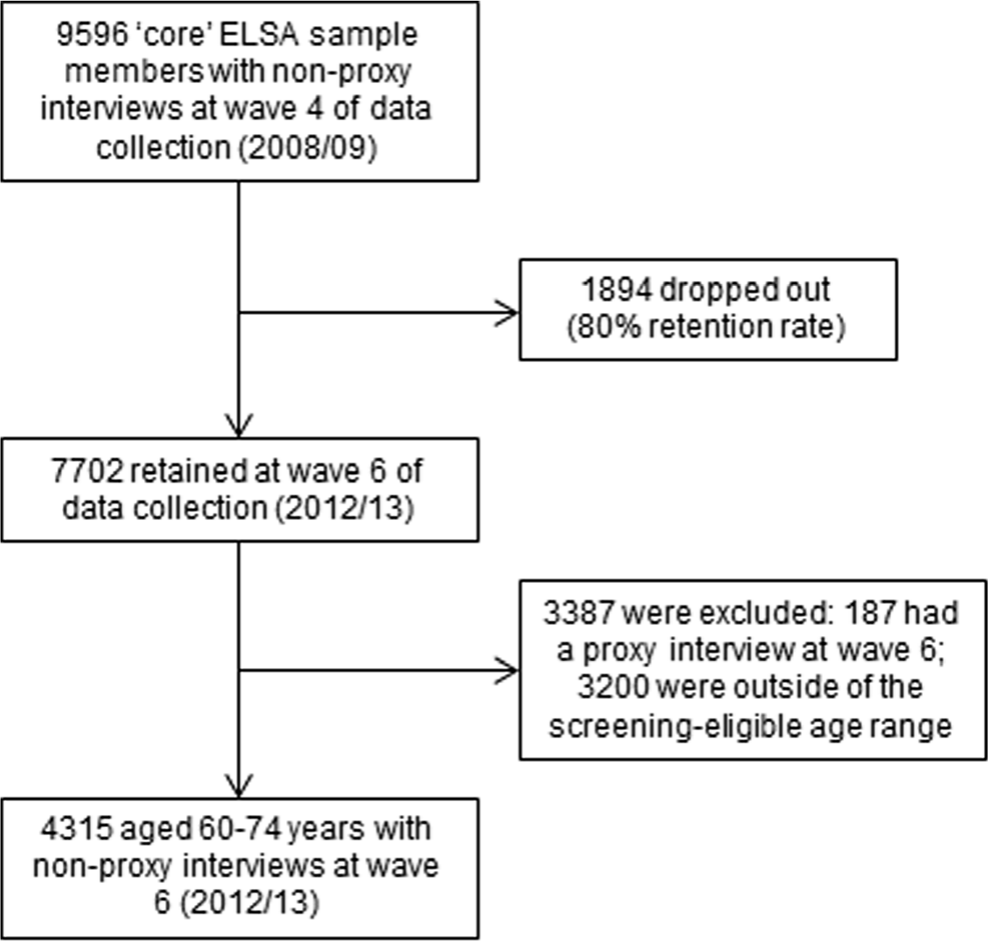
Study eligibility flow

The present analysis uses data from waves 4 (2008/2009) and 6 (2012/2013) of the ELSA data collection. Perceived life expectancy was assessed in the study interview in 2008/2009 (wave 4; “baseline”), and completion of at least one FOBt through the NHS Bowel Cancer Screening Programme from 2010 to 2012/2013 was assessed in the study interview in 2012/2013 (wave 6; ‘follow-up’). Eligible respondents for this analysis were core ELSA sample members who were aged 60–74 years at the follow-up (i.e., within the FOBt screening-eligible age range) with non-proxy study interviews. Proxy interviews with a family member or friend were conducted if the respondent was cognitively impaired or institutionalized. Of the 9896 core ELSA sample members in 2008/2009, 300 had a proxy interview and were excluded, leaving 9596 potentially eligible participants. Of these, 7704 were retained in 2012/2013, giving an 80% retention rate over the 4-year follow-up period. Of these, 187 had a proxy interview at wave 6 and a further 3200 were outside of the eligible age range for FOBt screening. In total, the eligible sample for this analysis comprised 4315 men and women (Fig. 1).

### Ethical Approval and Participant Consent

The ELSA was approved by the London Multicentre Research Ethics Committee (MREC/01/2/91), and informed consent was obtained from all participants.

### Measures

Perceived life expectancy was assessed in the study interview by the question, “*What are the chances (from 0%-100%) you will live to be age X or more?*” *X* was defined as follows: if the participant was age 65 or under, *X* = 75; if age 66–69, *X* = 80; and if age 70–74, *X* = 85. The participants were presented with a show card displaying a scale from 0 to 100 to aid their responses.

Uptake of FOBt-based CRC screening in England’s NHS Bowel Cancer Screening Programme was assessed in the study interview by the question, “*Have you ever completed a home testing kit for screening bowel cancer?*” (yes, no). Those who responded “yes” were then asked, “*How long ago was your most recent test?*” (month, year), and “*Was this test part of the NHS Bowel Cancer Screening Programme?*” (yes, no). Those who responded that their most recent test was prior to 2010 were coded as “no” for uptake of screening over the study follow-up (*n* = 151; a small number as the screening program was not fully implemented nation-wide until 2010). Another small number reported that their test was not part of the screening program (*n* = 54). Self-reported uptake of FOBt in the context of England’s national program has recently been shown in a similar population-based sample to be valid against NHS uptake records, with 94.2% agreement and *κ* = 0.74 [15].

Covariates assessed in the study interview that are known to be, or are plausibly, associated with one or both of PLE and CRC screening were age (continuous), sex, educational attainment (post-secondary degree or equivalent; up to a post-secondary degree; no qualifications [including no high school qualification]), ethnicity (white; non-white), marital status (married or living as married; single), smoking status (never; former; current), self-rated health (excellent; very good; good; fair; poor), and previously having had a diagnosis of cancer, cardiovascular disease, or high blood pressure (no; yes). Ages of mother and father currently or at death (continuous; per 10 years) were included as a proxy for health endowment, as people may predict their own life expectancies informed by those of their parents [13].

Because PLE is on a numeric scale, numeracy was measured in the study interview using six basic questions such as, ‘*If the chance of getting a disease is 10 percent, how many people out of 1,000 (one thousand) would be expected to get the disease?*” (answer options: 100; 10; 90; 900). Each question was scored as correct or incorrect, and they were summed to give a total score out of six. The continuous numeracy score was included in this analysis, to account for any numeracy difficulties that might affect response to the PLE question.

### Statistical Analysis

PLE was categorized into low (<25%), middle low (25–49%), middle high (50–74%), and high (≥75%) chance of surviving another 10–15 years. This categorization had the advantage of smoothing out focal points in responses and therefore reducing the likelihood of focal response bias in the results. The focal response bias is a cognitive bias, where people will round a continuous numerical estimate to the nearest whole number (in this case, at intervals of 10 on a scale of 0 to 100), rather than responding on a truly continuous scale, as the probability of survival should be in reality [11, 16]. The most common focal points we observed were at 50, 80, and 70%, with smaller spikes seen at other 10-year intervals, consistent with PLE data observed elsewhere [13].

Characteristics of the sample (PLE and covariates) were described according to FOBt uptake status (defined as yes vs. no for completion of at least one FOBt over the follow-up period). Means and standard deviations for continuous variables were compared across FOBt uptake status using the Wilcoxon rank-sum test. Frequency counts for categorical variables were compared across FOBt uptake status using the chi-squared test. Logistic regression was used to estimate odds ratios (ORs) and 95% confidence intervals (CIs) for the prospective association between PLE and FOBt uptake status. Both unadjusted and fully adjusted models including all covariates were estimated. The final analytical sample contained *n* = 3975 participants, as 39 (<1%) were missing data on PLE, 8 (<1%) on education, 1 (<1%) on ethnicity, 2 (<1%) on self-rated health, 132 (3%) on mother’s age currently or at death, 233 (5%) on father’s age currently or at death, and 12 (<1%) on numeracy.

To examine the accuracy of PLE, we compared PLE responses with objectively estimated 10-year mortality risk according to the ELSA Mortality Risk Index [17]. The ELSA Mortality Risk Index was developed and validated in the baseline ELSA sample and is harmonized with a similar index developed for the US Health and Retirement Study (HRS) [18, 19]. The ELSA Mortality Risk Index assigns a risk score to individuals based on age, sex, comorbid conditions (cancer, lung disease, and heart failure), smoking status, level of physical activity, and difficulty with functional tasks (preparing a hot meal, walking 100 yards, and pushing and pulling large objects). The risk scores correspond to a 10-year risk for all-cause mortality [17]. We compared mean PLE estimates across the risk scores. To assess the relationship between PLE and FOBt screening independently of estimated 10-year mortality risk, the fully adjusted model from above was additionally adjusted for ELSA Mortality Index score and the covariates that were not included within the index (education, ethnicity, marital status, self-rated health, numeracy, and ages of mother and father currently or at death).

Sensitivity analyses were performed removing those who reported completing an FOBt kit outside of the national program (*n* = 54) and removing those who reported that their most recent FOBt was prior to 2010 (*n* = 151). We also restricted the analysis to those whose PLE estimates were 10 to 11 years in the future, rather than 10 to 15 years into the future, to examine whether the prediction timespan affected the results. All analyses were conducted at the 95% confidence level using StataSE 13.1 (College Station, Texas).

## Results

Table 1 shows the baseline characteristics of participants. Grouping the PLE responses, 6% of participants (243/3975) estimated their chance of living another 10–15 years as 0–24%, 8% (312/3975) estimated a 25–49% chance, 44% (1737/3975) estimated a 50–74% chance, and 42% (1683/3975) estimated a 75–100% chance. The mean age at baseline was 62.6 years (SD 4.1). Just over half of participants were female (55%; 2174/3975). Nearly one third had post-secondary degree education (29%; 1152/3975) and 22% had no educational qualifications (885/3975). Only 2% of the sample was not white (88/3975). Most participants were married or living as married (77%; 3071/3975). On average, the age of mother currently or at death was 78 years (SD 12), and the age of father currently or at death was 72 years (SD 13). Smoking status, self-rated health, and chronic disease diagnoses are also shown in Table 1.

**Table 1:** Baseline sample characteristics, overall and according to FOBt screening, The English Longitudinal Study of Ageing, 2008–13, *n* = 3975

Baseline characteristic	Overall *N* (%)	FOBt screening from 2010 to 2012		
		Yes 2817 (71%)	No 1158 (29%)	*p* value
Perceived life expectancy				
Low (0 to 24%)	243 (6%)	126 (5%)	117 (10%)	<0.0001
Lower middle (25 to 49%)	312 (8%)	197 (7%)	115 (10%)	
Higher middle (50 to 74%)	1737 (44%)	1222 (43%)	515 (45%)	
High (75 to 100%)	1683 (42%)	1272 (45%)	411 (35%)	
Age				
Mean SD	62.6 (4.1)	62.3 (3.9)	63.1 (4.6)	<0.0001
Sex				
Male	1801 (45%)	1238 (44%)	563 (49%)	0.007
Female	2174 (55%)	1579 (56%)	595 (51%)	
Educational attainment				
Post-secondary degree equivalent	1152 (29%)	851 (30%)	301 (26%)	<0.0001
Up to a post-secondary degree	1938 (49%)	1417 (50%)	521 (45%)	
No qualifications	885 (22%)	549 (20%)	336 (29%)	
Ethnicity				
White	3887 (98%)	2760 (98%)	1127 (97%)	0.21
Non-white	88 (2%)	57 (2%)	31 (3%)	
Marital status				
Married or living as married	3071 (77%)	2249 (80%)	822 (71%)	<0.0001
Single	904 (23%)	568 (20%)	336 (29%)	
Smoking status				
Never smoker	1574 (40%)	1175 (42%)	399 (34%)	<0.0001
Former smoker	1850 (47%)	1335 (47%)	515 (44%)	
Current smoker	551 (14%)	307 (11%)	244 (22%)	
Age of mother currently or at death				
Mean (SD)	78 (12)	78 (12)	77 (13)	0.03
Age of father currently or at death				
Mean (SD)	72 (13)	73 (13)	71 (14)	0.002
Numeracy score				
Mean (SD)	4.4 (1.2)	4.5 (1.2)	4.2 (1.3)	<0.0001
Self-reported general health				
Excellent	571 (14%)	426 (15%)	145 (13%)	<0.0001
Very good	1311 (33%)	989 (35%)	322 (28%)	
Good	1248 (31%)	889 (32%)	359 (31%)	
Fair	652 (16%)	413 (15%)	239 (21%)	
Poor	193 (5%)	100 (3%)	93 (8%)	
Cancer				
No	3699 (93%)	2630 (94%)	1069 (92%)	0.24
Yes	276 (7%)	187 (6%)	89 (8%)	
Cardiovascular disease				
No	3044 (77%)	2193 (78%)	851 (73%)	0.003
Yes	931 (23%)	624 (22%)	307 (27%)	
High blood pressure				
No	2444 (61%)	1765 (63%)	679 (59%)	0.02
Yes	1531 (39%)	1052 (37%)	479 (41%)	

Table 1 also shows the baseline characteristics of participants according to FOBt uptake status over the follow-up. Just over two thirds of participants (71%; 2817/3975) reported completing at least one FOBt over the follow-up. FOBt uptake greatly differed across PLE categories, with 52% (126/243) of participants who estimated a 0–24% PLE having completed the test compared to 76% (1272/1683) of those who estimated a 75–100% PLE (*p* < 0.0001). Most other covariates were, as predicted, significantly positively associated with FOBt uptake over the study follow-up period, including having an older age of either mother or father currently or at death and having a higher score on the numeracy assessment (Table 1). Adjusted analyses are therefore essential to examine the independent prospective association between PLE and FOBt uptake. Having a previous cancer diagnosis was not associated with FOBt uptake over the follow-up.

In the fully adjusted model (Table 2), PLE was associated with FOBt uptake in a statistically significant linear dose-response fashion (*p*_trend_ < 0.0001), with the following ORs predicting FOBt uptake (yes vs. no): OR = 1.32 (95% CI 0.93–1.88) for 25–49%, OR = 1.52 (95% CI 1.14–2.03) for 50–74%, and OR = 1.74 (95% CI 1.29–2.34) for 75–100%, all vs. 0–24% (Table 2). Older age was negatively associated with FOBt uptake (OR = 0.96; 95% CI 0.95–0.98 per year), as was having no educational qualifications (OR = 0.79; 95% CI 0.64–0.98 vs. degree level education), being single (OR = 0.72; 95% CI 0.61–0.84 vs. married or living as married), being a current smoker (OR = 0.52; 95% CI 0.42–0.65), and having poor self-rated health (OR = 0.64; 95% CI 0.44–0.92; Table 2). Being female was positively associated with FOBt uptake (OR = 1.30; 95% CI 1.12–1.51), as was having better numeracy (OR = 1.06; 95% CI 1.00–1.13 per point out of 6; Table 2). In the model adjusted for continuous ELSA Mortality Risk Index score and covariates, the relationship between PLE and FOBt screening became slightly stronger than in the previous ORs: OR = 1.35 (95% CI 0.93–1.88) for 25–49%, OR = 1.56 (95% CI 1.18–2.08) for 50–74%, and OR = 1.81 (95% CI 1.35–2.43) for 75–100%, all vs. 0–24% (Table 2). Independently of PLE and covariates, higher 10-year mortality risk according to the Index was associated with lower odds of participating in FOBt screening (OR = 0.91; 95% CI 0.88–0.94 per point increase; Table 2).

**Table 2:** Logistic regression models predicting FOBt screening, The English Longitudinal Study of Ageing, 2008–13, *n* = 3975

	Unadjusted OR (95% CI)	Adjusted OR 1^a^ (95% CI)	Adjusted OR 2^a^ (95% CI)
Perceived life expectancy			
Low (0 to 24%)	1.00 (ref)	1.00 (ref)	1.00 (ref)
Lower middle (25 to 49%)	1.59 (1.13, 2.24)	1.32 (0.93, 1.88)	1.33 (0.93, 1.88)
Higher middle (50 to 74%)	2.20 (1.68, 2.89)	1.52 (1.14, 2.03)	1.57 (1.18, 2.08)
High (75 to 100%)	2.87 (2.18, 3.78)	1.74 (1.29, 2.34)	1.81 (1.35, 2.43)
Age			
Per year	0.95 (0.94, 0.97)	0.96 (0.95, 0.98)	–
Sex			
Male	1.00 (ref)	1.00 (ref)	–
Female	1.20 (1.05, 1.38)	1.30 (1.12, 1.51)	–
Educational attainment			
Post-secondary degree or equivalent	1.00 (ref)	1.00 (ref)	1.00 (ref)
Up to a post-secondary degree	0.96 (0.82, 1.14)	1.04 (0.88, 1.23)	1.03 (0.87, 1.22)
No qualifications	0.58 (0.48, 0.70)	0.79 (0.64, 0.98)	0.79 (0.64, 0.97)
Ethnicity			
White	1.00 (ref)	1.00 (ref)	1.00 (ref)
Non-white	0.75 (0.48, 1.17)	0.83 (0.52, 1.32)	0.85 (0.53, 1.35)
Marital status			
Married or living as married	1.00 (ref)	1.00 (ref)	1.00 (ref)
Single	0.62 (0.53, 0.72)	0.72 (0.61, 0.84)	0.71 (0.60, 0.83)
Smoking status			
Never smoker	1.00 (ref)	1.00 (ref)	–
Former smoker	0.88 (0.76, 1.03)	0.94 (0.81, 1.10)	–
Current smoker	0.43 (0.35, 0.52)	0.52 (0.42, 0.65)	–
Age of mother currently or at death			
Per 10 years	1.06 (1.01, 1.12)	1.00 (0.95, 1.06)	1.01 (0.95, 1.07)
Age of father currently or at death			
Per 10 years	1.08 (1.03, 1.14)	1.04 (0.98, 1.10)	1.04 (0.99, 1.10)
Numeracy score			
Per 1 point (out of 6)	1.16 (1.10, 1.23)	1.06 (1.00, 1.13)	1.05 (0.99, 1.12)
Self-reported general health			
Excellent	1.00 (ref)	1.00 (ref)	1.00 (ref)
Very good	1.05 (0.83, 1.31)	1.12 (0.89, 1.41)	1.12 (0.89, 1.41)
Good	0.84 (0.67, 1.06)	0.99 (0.79, 1.26)	1.02 (0.81, 1.29)
Fair	0.59 (0.46, 0.75)	0.86 (0.66, 1.13)	0.93 (0.71, 1.21)
Poor	0.37 (0.26, 0.51)	0.64 (0.44, 0.92)	0.75 (0.51, 1.08)
Cancer			
No	1.00 (ref)	1.00 (ref)	–
Yes	0.85 (0.66, 1.11)	0.90 (0.68, 1.19)	–
Cardiovascular disease			
No	1.00 (ref)	1.00 (ref)	–
Yes	0.79 (0.67, 0.92)	1.00 (0.84, 1.19)	–
High blood pressure			
No	1.00 (ref)	1.00 (ref)	–
Yes	0.84 (0.73, 0.97)	0.98 (0.84, 1.14)	–
ELSA Mortality Risk Index score^b^			
Per 1 point (out of 11)	0.87 (0.84, 0.89)	–	0.91 (0.88, 0.94)

^a^Adjusted for all variables in the column

^b^ELSA Mortality Risk Index scores include age, sex, comorbid conditions (cancer, lung disease, and heart failure), smoking status, level of physical activity, and difficulty with functional tasks (preparing a hot meal, walking 100 yards, and pushing and pulling large objects). Scores ranged from 1 to 18 points, but are collapsed at 11 points

Table 3 shows the mean PLE estimates across each scoring point on the ELSA Mortality Risk Index. The continuous PLE estimates decreased linearly as ELSA Index scores increased, with a correlation of moderate strength (Pearson’s *r* = −0.27; *p* < 0.0001). In this study, the ELSA Index scores ranged from 1 point (lowest mortality risk, corresponding to a 10-year mortality rate of 3% in the development study) to 18 points (highest mortality risk, corresponding to a 10-year mortality rate of 95% in the development study). In those who scored 1 point on the ELSA Index, the mean self-reported chance of living another 10–15 years was 73.6% (SD 17.9%). In those who scored 12 or more points on the ELSA Index, the mean self-reported chance of living another 10–15 years was 46.4% (SD 27.4%).

**Table 3:** PLE according to the ELSA Index for 10-year mortality risk

ELSA Index point score	Number	Mean PLE (SD)
1	239	73.6 (17.9)
2	299	71.4 (17.8)
3	608	71.4 (19.8)
4	568	67.8 (21.3)
5	626	67.5 (20.7)
6	585	64.0 (22.0)
7	432	62.2 (24.9)
8	298	57.4 (24.4)
9	132	52.4 (26.9)
10	110	52.8 (27.2)
≥11	78	43.7 (28.0)

The main results were not affected in the sensitivity analyses excluding from the fully adjusted model each of the 54 participants who reported completing the FOBt outside the national screening program (OR = 1.31; 95% CI 0.92–1.87 for 25–49%, OR = 1.48; 95% CI 1.11–1.97 for 50–74%, OR = 1.69; 95% CI 1.26–2.29 for 75–100%, all vs. 0–24%) and the 151 participants who reported completing FOBt screening prior to the study period (OR = 1.40; 95% CI 0.97–2.03 for 25–49%, OR = 1.53; 95% CI 1.13–2.06 for 50–74%, OR = 1.76; 95% CI 1.29–2.40 for 75–100%, all vs. 0–24%). Restricting the analysis to those whose PLE estimates ranged from 10 to 11 years, rather than 10 to 15 years, as in the full sample, resulted in a slightly stronger magnitude of association between PLE and screening uptake (not shown).

## Discussion

In this population-based cohort study of English men and women who were age-eligible for nationally organized FOBt-based CRC screening, perceived life expectancy at baseline was a strong positive predictor of FOBt uptake over the 4-year study follow-up. Three quarters of men and women who rated their chances of living another 10 to 15 years as 75–100% at baseline completed at least one FOBt over the follow-up, compared to half of those who rated their chances as 0–24%. This finding was independent of sociodemographic factors, numeracy skills, self-rated health, and other risk factors for mortality, including smoking status and chronic disease diagnoses. It was also independent of score on a validated index of 10-year mortality risk for older adults, indicating that perceived and objectively estimated mortality risk might make unique contributions to predicting CRC screening behavior. Our finding has particular salience for two issues in CRC screening: the fact that CRC screening is not recommended for adults with an actual life expectancy of less than 10 years, and persistent socioeconomic inequalities in CRC screening uptake.

Based on increasing evidence for the lack of benefit of CRC screening in older adults with a low life expectancy, the American College of Physicians does not recommend screening in adults with less than 10 years of remaining actual life expectancy [12]. In this group, the benefits of early diagnosis are minimal and considered to be outweighed by the potential harms of screening including unnecessary diagnostic procedures and overtreatment. In the USA, recent studies have found that participation in cancer screening is common among this group [20–22], sparking the development of strategies to tailor screening guidelines to account for differential life expectancies within age strata [23, 24]. Uptake of screening among adults with low life expectancy is thought to occur partly because there is generally high public enthusiasm for cancer screening at older ages and also because the contraindication of screening can be difficult for physicians to communicate to patients with high mortality risk [25–28]. Information about the time frame that elapses before screening confers a mortality benefit is not communicated to the public as part of the screening invitations in the UK, and it does currently not form part of the guidelines for CRC screening given by the US Centers for Disease Control and Prevention, the US Preventive Services Task Force, or the American Cancer Society [28–30].

Better public communication about the aims of screening may help to optimize uptake, as our study indicates that people may take into account their expectations of the future when making decisions about screening. This is good news, although a substantial proportion of adults who perceived their own life expectancy as low—less than a 25% chance of living another 10–15 years—still participated in CRC screening. Assuming a reasonable level of accuracy in their life expectancy estimations, the reasons why these adults complete screening despite their low expectations of future survival require further investigation. Conversely, many people with high expectations of their future survival did not complete CRC screening and consequently may be missing out on the benefits of screening intended for this group. These findings are concerning, as PLE showed good concordance with objectively estimated 10-year mortality risk in this study and was predictive of actual mortality risk in a national sample of older American adults. In the HRS, older adults who survived over a 2-year follow-up had, on average, a 50-point higher estimate of 15–25-year PLE than those who died [10]. In a longer 8-year follow-up, those who died reported a 56% chance of 15–25-year survival at baseline, on average, compared with a 65% average chance reported by those who survived [11].

The potential impact of life expectancy perceptions on socioeconomic inequalities in CRC screening uptake is also important to consider. Inequalities in screening uptake based on several socioeconomic indicators have been demonstrated in several country contexts [31, 32]. Adults with low educational attainment and who have experienced economic hardship have, on average, lower expectations for their remaining life expectancy than those who are better off [33]. Having higher perceived life expectancy is thought to increase one’s sense of control over their own life and events in the future (the “horizon hypothesis”) [34], which could in turn increase the likelihood of participating in cancer screening [35]. Low perceptions of personal life expectancy, if more frequent among adults of low socioeconomic status, might have the adverse effect of contributing to inequalities in CRC screening uptake and eventually to cancer outcomes, including mortality. Further work is needed to examine the accuracy of life expectancy perceptions across socioeconomic groups, in order to establish the degree to which lowered expectations of the future may present a barrier to cancer screening that could be targeted and modified by public communication strategies.

Our results are consistent with those from the European SHARE study, which found that PLE was positively associated with mammography uptake in women [13]. In our study, “ever” FOBt uptake over the follow-up from 2010 to 2012/2013 was 71%, consistent with NHS records for ever uptake of 70.1% in the general eligible population of over 60,000 men and women in the southern region of England over a similar time period [36]. Overall, uptake was negatively associated with age in our study. When screening was initially available to 60–69-year-olds from 2006 to 2010, age was positively associated with uptake, likely because older adults would have received more biennial screening invitations than younger adults within this age range [36]. Adults aged 70–74 years were then invited from 2010 as part of an age extension. In the period from 2006 to 2012, NHS records show that uptake peaks in the middle of the 60–74 age range [36]. Our results also show this trend when broken down by age at follow-up, with uptake at 64% among 60-year-olds, 75% among 69-year-olds, and 50% among 74-year-olds. Our results for the other predictors of FOBt uptake, which were being female, having higher education, being married, not smoking, having better numeracy skills, and better self-rated health, are consistent with previous research [31, 32, 36–39].

A limitation of this study is that FOBt uptake was assessed through self-report. However, uptake rates were consistent with those observed in the general screening-eligible population over a similar time period [36]. Self-reported screening uptake in a similar sample, in the context of England’s NHS Bowel Cancer Screening Programme, was recently highly concordant with NHS records (94.2% agreement, *κ* = 0.74) [15]. History of colonoscopy or sigmoidoscopy or completion of a physician-recommended FOBt was not assessed in the study interview. These scenarios, respectively, would have made participants ineligible for the program or may have overridden any effects of PLE on screening behavior. We expect that this would apply to the minority of study participants. We did not have data on the total number of FOBt screening kits that a person had completed in the past. Some people in the sample may have completed screening prior to the study period, as the screening program was implemented in small areas in 2006 and rolled out nationwide during the period 2006 to 2010 [40]. The reverse relationship of screening participation on PLE is uncertain and may depend on the time elapsed between a screening episode and the PLE assessment. The PLE estimates in this study may represent, to a degree, the aggregate of health-related behaviors and events that people experienced over their lives. When considered in this way, there may be bidirectional relationships between PLE and health-related behaviors, a question that requires further investigation.

Although the ELSA aims to be population-representative, it is subject to non-response and attrition bias, as with any longitudinal cohort study. Compared with participants who remained in the study, those who dropped out between waves 4 and 6 had lower educational attainment, were more socioeconomically deprived, and were in worse health (self-rated and diagnoses of cardiovascular disease and cancer). They were underrepresented in this analysis. Mean PLE was also lower among those who dropped out of the study than those who remained in the study (51.4%, SD 30.9 vs. 59.9%, SD 26.6%; *p* < 0.0001). Consequently, if those who dropped out were less likely to have participated in FOBt screening than those who remained in, we may have underestimated the true association between PLE and screening uptake. Only 2% of participants were non-white, so we did not have adequate statistical power to examine the role of ethnicity.

Measurement error may have been introduced if participants’ PLE estimates changed between the study baseline and the actual time of cancer screening. If this occurred and was non-differential across the study population, then odds ratios for PLE and FOBt screening will be biased towards the null, underestimating the magnitude of the true association. If certain subgroups were more likely to decrease or increase in PLE than others, the odds ratios may be biased downwards or upwards. If PLE estimates were relatively accurate, then they would, to a degree, represent the same underlying construct as objectively estimated mortality risk (i.e., actual mortality risk). The PLE estimates showed good concordance with 10-year mortality risk according to the validated and reliable ELSA Mortality Risk Index [17]. While our results indicate that there is overlap between self-perceived and objectively estimated mortality risk, they predicted FOBt uptake independently of one another, indicating that they might make unique contributions to predicting behavior. We hypothesize that inaccuracies in PLE estimates would be greatest among those older adults with low educational attainment, low socioeconomic status, or poor literacy or numeracy skills or who have experienced aging-related cognitive decline. Further work with follow-up mortality data is needed to identify which adults had incorrectly low or high perceptions of their remaining life expectancy. The two scenarios may have different implications for cancer screening and potentially other health behaviors as well. This is an important area for future work.

In summary, this is the first longitudinal analysis of perceived life expectancy and CRC screening uptake in the context of an organized, publicly available screening program. We found that men and women who rated their chance of living another 10 to 15 years as 75–100% at baseline were significantly more likely to complete at least one FOBt for CRC screening than those who rated their chance as 0–25%. A substantial proportion of adults with low expectations of survival participated in CRC screening, despite this screening test not being recommended for adults with low remaining life expectancy. If PLE estimations are reasonably accurate among this group, they might be subject to greater harm than benefit from screening. At the same time, one quarter of adults with high expectations of their own life expectancy appeared to be missing out on the benefits of screening intended for this group. It appears that CRC screening may not be optimized to the greatest possible benefit for either group. A better understanding of how people develop their mental models of PLE might help identify those who are at risk of overly fatalistic attitudes towards their futures and potentially at risk of declining important health services such as cancer screening.

While we do not recommend that PLE be used as a basis for decision making about screening, further research should investigate whether public communication about the role of actual life expectancy in conferring the benefits of cancer screening might help to improve decision making. Any potential communication strategies would have to be carefully designed and implemented to avoid any public misunderstanding. Mortality risk calculators that are validated, accurate, and suitably brief could be useful tools to aid in shared clinical decision making with respect to cancer screening, although their potential to foster fatalistic attitudes about life expectancy must be carefully considered. Finally, if the accuracy of life expectancy perceptions differs across population subgroups, the implications of public communication for social equity in uptake of screening will require careful consideration.

## References

[1] Torre LA, Bray F, Siegel RL, Ferlay J, Lortet-Tieulent J, Jemal A (2015). Global cancer statistics, 2012. CA Cancer J Clin..

[2] Schreuders EH, Ruco A, Rabeneck L (2015). Colorectal cancer screening: A global overview of existing programmes. Gut..

[3] Maringe C, Walters S, Rachet B (2013). Stage at diagnosis and colorectal cancer survival in six high-income countries: A population-based study of patients diagnosed during 2000-2007. Act Oncol..

[4] Hardcastle JD, Chamberlain JO, Robinson MH (1996). Randomised controlled trial of faecal-occult-blood screening for colorectal cancer. Lancet..

[5] Mandel JS, Church TR, Ederer F, Bond JH (1999). Colorectal cancer mortality: Effectiveness of biennial screening for fecal occult blood. J Natl Cancer Inst..

[6] Carstensen LL (2006). The influence of a sense of time on human development. Science..

[7] Hamermesh DS (1985). Expectations, life expectancy, and economic behavior. Q J Econ..

[8] Ehrlich I, Chuma H (1990). A model of the demand for longevity and the value of life extension. J Polit Econ..

[9] Hurd MD, McGarry K (2002). The predictive validity of subjective probabilities of survival. Econ J..

[10] Smith VK, Taylor DH, Sloan FA (2001). Longevity expectations and death: Can people predict their own demise?. Am Econ Rev..

[11] Hurd MD (2009). Subjective probabilities in household surveys. Annu Rev Econ..

[12] Qaseem A, Denberg TD, Hopkins RH (2012). Screening for colorectal cancer: A guidance statement from the American College of Physicians. Ann Intern Med..

[13] Wuebker A (2012). Who gets a mammogram amongst European women aged 50-69 years?. Health Econ Rev..

[14] Steptoe A, Breeze E, Banks J, Nazroo J (2013). Cohort profile: The English longitudinal study of ageing. Int J Epidemiol..

[15] Lo SH, Waller J, Vrinten C, Wardle J, von Wagner C (2016). Self-reported and objectively recorded colorectal cancer screening in England. J Med Screen..

[16] Hurd M, McFadden D, Gan L, Wise D (1998). Subjective survival curves and life cycle behavior. Inquiries in the Economics of Aging.

[17] Kobayashi LC, Jackson SE, Lee SJ, Wardle J, Steptoe A. The development and validation of an index to predict 10-year mortality risk in a longitudinal cohort of older English adults. *Age Ageing* (forthcoming). 10.1093/ageing/afw199 PMC5405757 27810854

[18] Lee SJ, Lindquist K, Segal MR, Covinsky KE (2006). Development and validation of a prognostic index for 4-year mortality in older adults. JAMA..

[19] Cruz M, Covinsky KE, Widera EW, Stijacic-Cenzer I, Lee SJ (2013). Accurately predicting 10-year mortality for older Americans: An extension of the Lee Index. JAMA..

[20] Schonberg MA, Breslau ES, Hamel MB, Bellizzi KM, McCarthy EP (2015). Colon cancer screening in U.S. adults aged 65 and older according to life expectancy and age. J Am Ger Soc..

[21] Schonberg MA, Breslau ES, McCarthy EP (2013). Targeting of mammography screening according to life expectancy in women aged 75 and older. J Am Ger Soc..

[22] Royce TJ, Hendrix LH, Stokes WA, Allen IM, Chen RC (2014). Cancer screening rates in individuals with different life expectancies. JAMA Intern Med..

[23] Cho H, Klabunde CN, Yabroff KR, Wang Z, Meekins A (2013). Comorbidity-adjusted life expectancy: A new tool to inform recommendations for optimal screening strategies. Ann Intern Med..

[24] Lansdorp-Vogelaar I, Gulati R, Mariotto AB (2014). Personalizing age of cancer screening cessation based on comorbid conditions: Model estimates of harms and benefits. Ann Intern Med..

[25] von Wagner C, Macedo A, Campbell C (2013). Continuing cancer screening later in life: Attitudes and intentions among older adults in England. Age Ageing..

[26] Gross CP (2014). Cancer screening in older persons: A new age of wonder. JAMA Intern Med..

[27] Rochman S (2014). Cancer screening in older adults: Risks and benefits. J Natl Cancer Inst..

[28] Centers for Disease Control and Prevention. Colorectal cancer screening guidelines. Available at http://www.cdc.gov/cancer/colorectal/basic_info/screening/guidelines.htm. Accessed January 22, 2016.

[29] U.S. Preventive Services Task Force. Colorectal cancer: Screening. Available at http://www.uspreventiveservicestaskforce.org/Page/Document/UpdateSummaryFinal/colorectal-cancer-screening. Accessed January 22, 2016.

[30] American Cancer Society. American Cancer Society recommendations for colorectal cancer early detection. Available at http://www.cancer.org/cancer/colonandrectumcancer/moreinformation/colonandrectumcancerearlydetection/colorectal-cancer-early-detection-acs-recommendations. Accessed January 22, 2016.

[31] von Wagner C, Baio G, Raine R (2011). Inequalities in participation in an organized national colorectal cancer screening programme: Results from the first 2.6 million invitations in England. Int J Epidemiol..

[32] Meissner HI, Breen N, Klabunde CN, Vernon SW (2006). Patterns of colorectal cancer screening uptake among men and women in the United States. Cancer Epidemiol Biomarkers Prev..

[33] Mirowsky J, Ross CE (2000). Socioeconomic status and subjective life expectancy. Soc Psychol Q..

[34] Mirowsky J (1997). Age, subjective life expectancy, and the sense of control: The horizon hypothesis. J Gerontol B Psychol Sci Soc Sci..

[35] McQueen A, Tiro JA, Vernon SW (2008). Construct validity and invariance of four factors associated with colorectal cancer screening across gender, race, and prior screening. Cancer Epidemiol Biomarkers Prev..

[36] Lo SH, Halloran S, Snowball J, Seaman H, Wardle J, von Wagner C (2015). Colorectal cancer screening uptake over three biennial invitation rounds in the English bowel cancer screening programme. Gut..

[37] Kobayashi LC, Wardle J, von Wagner C (2014). Limited health literacy is a barrier to colorectal cancer screening in England: Evidence from the English Longitudinal Study of Ageing. Prev Med..

[38] Neter E, Stein N, Rennert G, Hagoel L. Self-rated health is prospectively associated with uptake of screening for the early detection of colorectal cancer, not vice versa. *Eur J Cancer Prev.* 2015; in press. 10.1097/CEJ.0000000000000184 26230609

[39] Blanks RG, Benson VS, Alison R (2015). Nationwide bowel cancer screening programme in England: Cohort study of lifestyle factors affecting participation and outcomes in women. Br J Cancer..

[40] Logan R, Patnick J, Nickerson C, Coleman L, Rutter MD, von Wagner C (2012). Outcomes of the Bowel Cancer Screening Programme (BCSP) in England after the first 1 million tests. Gut..

